# Combined analysis of PTEN, HER2, and hormone receptors status: remodeling breast cancer risk profiling

**DOI:** 10.1186/s12885-021-08889-z

**Published:** 2021-10-28

**Authors:** Elham Sajjadi, Konstantinos Venetis, Roberto Piciotti, Donatella Gambini, Concetta Blundo, Letterio Runza, Stefano Ferrero, Elena Guerini-Rocco, Nicola Fusco

**Affiliations:** 1 Department of Oncology and Hemato-Oncology, University of Milan, Via Festa del Perdono 7, 20122 Milan, Italy; 2 Division of Pathology, IEO, European Institute of Oncology IRCCS, Via Giuseppe Ripamonti 435, 20141 Milan, Italy; 3 Division of Medical Oncology, Fondazione IRCCS Ca’ Granda – Ospedale Maggiore Policlinico, Via Francesco Sforza 35, 20122 Milan, Italy; 4 Breast Surgery Unit, Fondazione IRCCS Ca’ Granda – Ospedale Maggiore Policlinico, Via Francesco Sforza 35, 20122 Milan, Italy; 5 Division of Pathology, Fondazione IRCCS Ca’ Granda – Ospedale Maggiore Policlinico, Via Francesco Sforza 35, 20122 Milan, Italy; 6 Department of Biomedical, Surgical, and Dental Sciences, University of Milan, Via della Commenda 10, 20122 Milan, Italy

**Keywords:** PTEN, Hormone receptors, Estrogen receptor, Progesterone receptor, HER2, Breast cancer, Prognosis, Biomarkers

## Abstract

**Background:**

Phosphatase and tensin homolog (PTEN) loss is associated with tumorigenesis, tumor progression, and therapy resistance in breast cancer. However, the clinical value of PTEN as a biomarker in these patients is controversial. We sought to determine whether the benefit of traditional biomarkers testing is improved by the analysis of PTEN status for the identification of high-risk breast cancer.

**Methods:**

A cohort of 608 patients with breast cancer was included in this study. Based on the expression on the neoplastic cells compared to the normal internal controls by immunohistochemistry (IHC), cases were classified as PTEN-low (PTEN-L) or PTEN-retained (PTEN-WT). The former constituted the study group, while the latter the control group. Analysis of gene expression was performed on publicly available genomic data and included 4265 patients from the METABRIC and MSK cohorts retrieved from cBioPortal. The Shapiro-Wilk test was used to analyze the normal distributions of continuous variables. Relationships between PTEN status and the clinicopathologic and molecular features of the patient population were assessed using Fisher’s exact test or Chi-squared/Wilcoxon rank-sum test. Survival curves were built according to the Kaplan-Meier method.

**Results:**

Alteration in PTEN status was significantly different at protein and gene levels, where the reduced protein expression was observed in 280/608 cases (46.1%) from our group, while genetic aberrations in only 315/4265 (7.4%) cases of the METABRIC and MSK cohorts. PTEN-L tumors were significantly enriched for hormone receptors (HR) and HER2 negativity (*n* = 48, 17.1%) compared to PTEN-WT tumors (*n* = 22, 6.7%; *p* = 0.0008). Lack of HR with or without HER2 overexpression/amplification was significantly associated with worse overall survival (OS) in PTEN-L but not in PTEN-WT breast cancers (*p* < .0001). Moreover, PTEN-L protein expression but not gene alterations was related to the outcome, in terms of both OS and disease-free survival (*p* = 0.002).

**Conclusions:**

The combined analysis of PTEN, HER2, and HR status offers relevant information for a more precise risk assessment of patients with breast cancer.

**Supplementary Information:**

The online version contains supplementary material available at 10.1186/s12885-021-08889-z.

## Background

Phosphatase and tensin homolog (PTEN) is a tumor suppressor and negative regulator of the phosphatidylinositol-3-kinase (PI3K)/protein kinase B (Akt) pathway [1, 2]. Loss of PTEN activity has been reported across a variety of primary and metastatic malignancies, including breast cancer, and is related to tumorigenesis, tumor progression, and therapy resistance [3–5].

In breast cancer, the clinical actionability of PTEN status has been studied in both prognostic and predictive settings [6, 7]. Alterations of PTEN and the serine/threonine kinase Akt isoforms have been observed in patients with HER2+ breast cancers with a low response to trastuzumab therapy [8]. Additionally, analysis of PTEN expression has been proposed as a complementary biomarker for mismatch repair status assessment in breast cancer, potentially contributing to the selection of patients, including those with a hormone receptor (HR) + tumor, eligible to immune-checkpoint blockade [4, 9]. Notably, the phase III Breast Cancer International Research Group (BCIRG)-006 trial demonstrated that PTEN loss is linked to a worse prognosis but not to trastuzumab resistance in patients with HER2+ breast cancer [10]. A meta-analysis of 27 studies including 10,231 breast cancers, further provided evidence that PTEN loss might be a predictor of aggressive behavior [11]. On the other hand, recent clinical and translational studies failed to identify a significant association between PTEN status and patients’ outcomes [12, 13]. So, the consistency of PTEN testing in clinical practice for patients with breast cancer remains unclear [14].

We hypothesized that, if alterations in PTEN have potent pro-oncogenic activity in breast cancer, detailed information on the status of this tumor suppressor could be used to improve clinical trial design and patients’ clinical management. In this study, we provide insights on the patterns of PTEN alterations along with HER2 and HR status in different groups of breast cancer and identify novel significant subsets of patients with high-risk neoplasms.

## Materials and methods

### Patients and tissue specimens

This study was approved by the local Ethical Committee under protocol number #620_2018bis. All patients included in this study were diagnosed and managed at the Fondazione IRCCS Ca′ Granda – Ospedale Maggiore Policlinico between 2004 and 2018, and they are part of an anonymized database encompassing detailed clinicopathologic and follow-up data [15]. For this study, patients were included based on the availability of formalin-fixed, paraffin-embedded (FFPE) and frozen tissue. All cases were reviewed, re-classified, and re-graded according to the latest World Health Organization (WHO) recommendations [16] and the Nottingham histologic grading system [17], respectively. Pathologic re-staging was performed following the 8th edition of the American Joint Committee on Cancer (AJCC) Cancer Staging Manual [18].

### Tissue microarrays construction

Representative FFPE blocks were selected for tissue microarray (TMA) construction, as previously described [19]. For each case, the core and periphery (i.e., invasive front) of the tumor, in situ (i.e., intraductal) component (if present), and matched normal epithelial breast tissue (i.e., glandular tissue with at least one non-neoplastic terminal ductal-lobular unit adjacent to the neoplasm) were sampled. A total of 5 tumor samples and one matched normal tissue per patient, with a diameter of 1 mm, were incorporated in the corresponding TMA block.

### Immunohistochemical analysis

Four-μm-thick sections were cut from the TMA blocks and subjected to immunohistochemistry (IHC) using anti-human pre-diluted antibodies for PTEN, estrogen receptor (ER), progesterone receptor (PgR), Ki67, and HER2 on a Dako Omnis automated staining systems (Agilent, Santa Clara, CA, USA) [20]. For each antibody, positive and negative controls were included in each slide run. HR (i.e. ER and PgR) and HER2 status were tested and reported according to the breast biomarker reporting guidelines v1.4.1.0 published by the College of American Pathologists (CAP) in June 2021 (available at https://www.cap.org/protocols-and-guidelines). The proliferation index was assessed by Ki67 IHC as the global (average) score across the section. According to the updated recommendations from the International Ki67 in Breast Cancer Working Group, a cut-off value of ≥30% was used to define the high proliferation group [21]. PTEN expression was scored using a three-tier system that considers the ratio between normal and tumor tissue, as proposed by Sakr et al. [22, 23]. Specifically, score 0 indicated the absence of staining in tumor cells but not in the surrounding normal epithelial and stromal cells, score 1 was considered when the tumor cell staining was weaker than the surrounding normal epithelial and stromal cells. In the case of staining equal to that of the normal epithelial and stromal cells, the case was scored as 2. Subsequently, PTEN status was assessed dichotomously as “low” (PTEN-L) and “wild type” (PTEN-WT) for cases with scores 0–1 and score 2, respectively [4]. Details of antibodies, clones, dilutions, antigen retrieval methods, and scoring systems adopted in this study are provided in Supplementary Table S1.

### cBioPortal and statistical analyses

Clinical and genomic data were extracted from the METABRIC and the MSK datasets made available by The Cancer Genome Atlas Network (TCGA) at cBioPortal [24]. Relationships between PTEN status and the clinicopathologic and molecular features of the patients were assessed using Fisher’s exact test or Chi-squared test [25]. Odds ratio (OR) and corresponding 95% confidence interval (CI) were calculated for each variable [26–28]. To identify factors associated with PTEN expression, multinomial logistic regression models were defined considering a stepwise selection procedure [29]. Survival curves were built according to the Kaplan-Meier method and compared using the log-rank test [30]. All statistical tests were two-tailed; *p*-values < 0.05 were considered statistically significant; reported *p*-values were not corrected for multiple testing.

## Results

A total of 608 patients with invasive breast cancer (age, 26–92 years; mean, 61.0 (12.9) years) diagnosed between 2004 and 2018 were included in this study (follow-up time, 1–172 months; mean, 57.8 (50.1) months). Their demographic and clinicopathologic characteristics are listed in Table 1. Follow-up data were available for 603 (99%) patients.

**Table 1:** Clinicopathologic features of the patients included in this study according to their biomarker status

	HR+/HER2-	HER2+	HR−/HER2-	Total
**All patients,** ***n*** **(%)**	488 (80)	50 (8)	70 (12)	608 (100)
**Age,** ***n*** **(%)**				
≥ 55 years	350 (83)	32 (8)	38 (9)	420 (69)
< 55 years	138 (73)	18 (10)	32 (17)	188 (31)
**Menopause,** ***n*** **(%)**				
Yes	383 (82)	41 (9)	44 (9)	468 (77)
No	103 (76)	8 (6)	25 (18)	136 (22)
n/a	2 (50)	1 (25)	1 (25)	4 (1)
**Histology,** ***n*** **(%)**				
Ductal	365 (79)	45 (10)	52 (11)	462 (76)
Lobular	71 (92)	3 (4)	3 (4)	77 (13)
Other	52 (75)	2 (3)	15 (22)	69 (11)
**Grade,** ***n*** **(%)**				
1	68 (94)	1 (1)	3 (4)	72 (12)
2	240 (93)	11 (4)	7 (3)	258 (42)
3	180 (65)	38 (14)	60 (22)	278 (46)
**ER,** ***n*** **(%)**				
Positive	488 (92)	40 (8)	0 (0)	528 (87)
Negative	0 (0)	10 (13)	70 (88)	80 (13)
**PgR,** ***n*** **(%)**				
Positive	418 (93)	30 (7)	0 (0)	448 (73)
Negative	70 (44)	20 (12)	70 (44)	160 (27)
**HER2,** ***n*** **(%)**				
Positive	0 (0)	50 (100)	0 (0)	50 (8)
Negative	488 (87)	0 (0)	70 (13)	558 (92)
**Ki67,** ***n*** **(%)**				
High	267 (72)	63 (11)	61 (17)	369 (61)
Low	221 (92)	9 (4)	9 (4)	239 (39)
**Stage,** ***n*** **(%)**				
I	228 (85)	19 (7)	21 (8)	268 (44)
II	172 (77)	15 (7)	35 (16)	222 (37)
III-IV	87 (74)	16 (14)	14 (12)	117 (19)
n/a	1 (100)	0 (0)	0 (0)	1
**Molecular subtype,** ***n*** **(%)**				
Luminal A^a^	204 (100)	0 (0)	0 (0)	204 (38)
Luminal B^b^	284 (88)	40 (12)	0 (0)	324 (49)
HER2-type1^c^	0 (0)	10 (100)	0 (0)	10 (2)
TNBC^d^	0 (0)	0 (0)	70 (100)	70 (11)

*HR* Hormone receptors, *ER* Estrogen receptor, *PgR* Progesterone receptor, *TNBC* Triple-negative breast cancer, *n/a* not available; ^a^ER+/PR+/Ki67 low; ^b^ER+/Ki67 high or ER+/PR-; ^c^ER−/PR−/HER2+; ^d^ER−/PR−/HER2-

### Decreased PTEN protein expression is more frequent than gene alterations in breast cancer

**Fig. 1:**
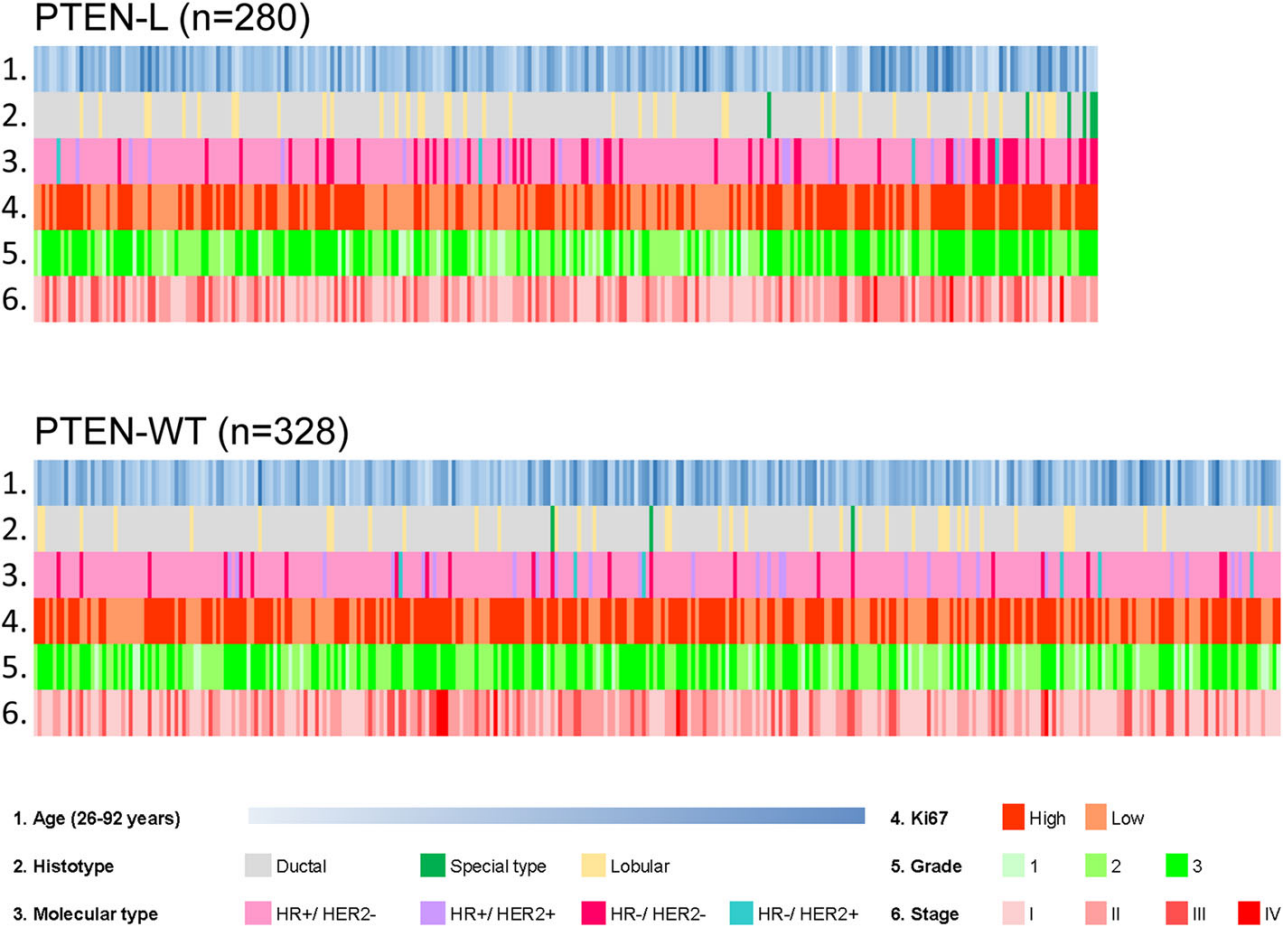
Heatmap illustrating selected clinicopathologic features of the patients included in this study according to the PTEN status. Each column represents a patient, each row a parameter, color-coded according to the legend below. PTEN-L, PTEN low (i.e. decreased expression); PTEN-WT, PTEN wild-type (i.e. retained expression) HR, hormone receptors

**Fig. 2:**
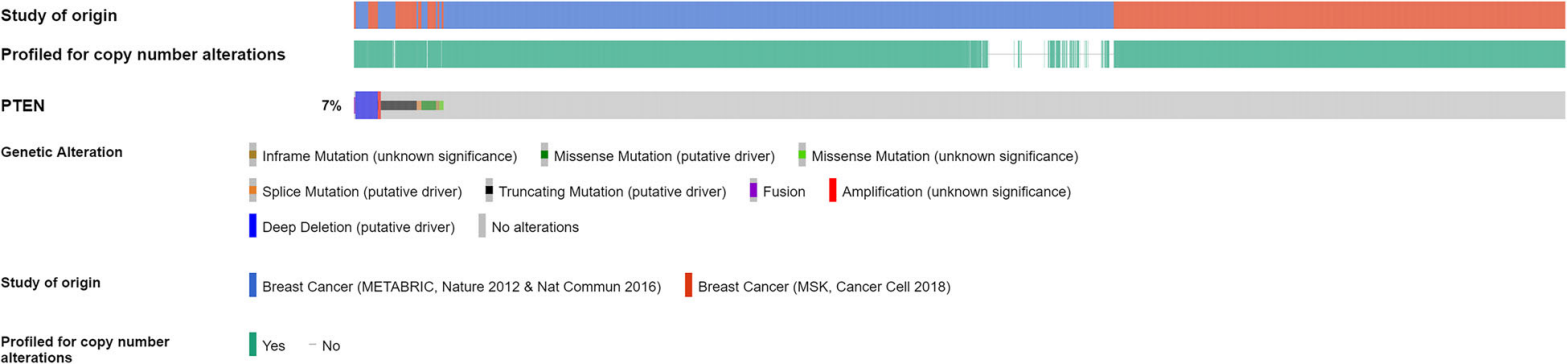
Oncoprint visualization of genetic alterations (i.e., inframe, missense, splice and truncating mutations, fusions, amplifications, and deep deletions) of the PTEN gene in breast cancer. In this analysis involving two different datasets available at cbioportal.org (patients *n* = 4265; samples *n* = 4427), truncating mutations and deep deletions were the most frequent alterations. Taken together, in 315 (7.4%) of the patients the queried gene was found to be altered. Each column represents a patient/sample and was sorted for the magnitude of alteration types in the queried genes. The types of alterations and the study of origin are color-coded as shown in the legend; the blue and red rectangles refer to the METABRIC and MSK studies, respectively

Taken together, 46.1% (*n* = 280/608) cases showed a decreased or null expression of the PTEN protein by IHC, as depicted in Fig. 1, and were therefore classified as PTEN-L. Conversely, analysis of the genomic data from the METABRIC and MSK portal cohorts including 4265 patients, revealed mutations, deep deletions, fusions, and/or amplifications in PTEN in only 315 (7.4%) patients (Fig. 2). These data suggest that, in breast cancer, alterations targeting PTEN are common events that more likely occur after transcription.

### The spectrum of PTEN alterations varies across different breast cancer subtypes

Among the patients included in this study, the mean age at diagnosis was similar in the PTEN-L (61.5 years) and PTEN-WT (60.6 years subgroups). In the former group, ductal (*n =* 224, 80%) and HR+/HER2- (*n =* 213, 76.1%) were the most frequent histological and molecular subtypes, respectively. Additionally, high grade (*n =* 130, 46.4%), and low stage (*n =* 118, 42.1%) tumors were more prevalent in the PTEN-L subgroup, as detailed in Fig. 1 and Table 2. Considering the PTEN intra-tumor expression pattern, the spatial distribution and IHC staining intensity were homogeneous, as demonstrated by the analysis of full sections in PTEN-L cases. On the other hand, a high degree of inter-tumor heterogeneity was observed, with a significant association between PTEN and HR/HER2 status (*p* = 0.0008 according to the chi-square independence test), as shown in Table 2). More in detail, in the PTEN-L population, the prevalence of HR−/HER2- tumors (*n =* 48, 17.1%) was higher compared to the PTEN-WT group (*n =* 22, 6.7%). These findings suggest that PTEN loss or reduced expression is a more common event in tumors lacking the HR and/or HER2 expression/amplification.

**Table 2:** Correlation between low and wildtype status of PTEN across selected clinicopathologic features

	PTEN-L	PTEN-WT	*p-*value
**All patients, n (%)**	280 (46.1)	328 (53.9)	
**Age, mean (SD)**	61.5 (12.0)	60.6 (13.6)	0.3425
**Histology, n (%)**			
Ductal	224 (80.0)	267 (81.4)	0.6243
Lobular	40 (14.3)	39 (11.9)	
Other	16 (5.7)	22 (6.7)	
**HR and HER2 status, n (%)**			
HR+/ HER2-	213 (76.1)	275 (83.8)	0.0008
HR−/ HER2+	4 (1.4)	6 (1,8)	
HR+/ HER2+	15 (5.4)	25 (7.6)	
HR−/ HER2-	48 (17.1)	22 (6.7)	
**Grade, n (%)**			
1	34 (12.1)	39 (11.9)	0.6428
2	116 (41.4)	148 (45.1)	
3	130 (46.4)	141 (43.0)	
**T, n (%)**			
1	180 (64.3)	198 (60.4)	0.7479
2	83 (29.6)	105 (32.0)	
3	6 (2.1)	8 (2.4)	
4	11 (3.9)	17 (5.2)	
**N, n (%)**			
Positive	164 (58.6)	207 (63.1)	0.2528
Negative	116 (41.4)	121 (36.9)	
**Stage, n (%)**			
0, 1	118 (42.1)	154 (47.0)	0.4682
2	107 (38.2)	112 (34.2)	
3, 4	55 (19.6)	62 (18.9)	

*PTEN-L* PTEN low (i.e. decreased expression), *PTEN-WT* PTEN wild-type (i.e. retained expression) *HR* Hormone receptors. *SD* Standard deviation

### PTEN status assessment improves HR and HER2 prognostic value

**Fig. 3:**
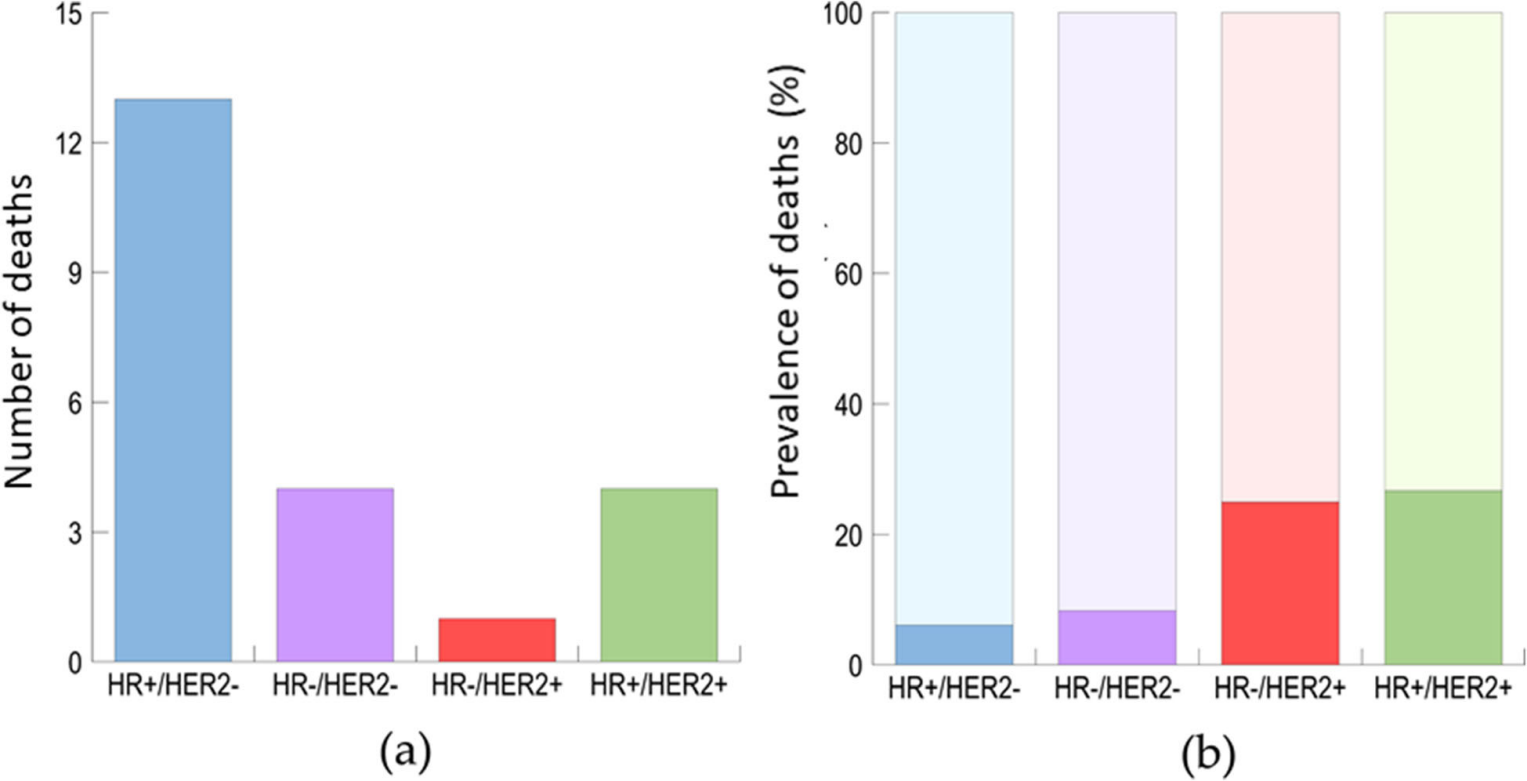
Frequency (**a**) and proportion (**b**) of death across PTEN-L breast cancer subgroups. ER, estrogen receptor; PTEN, phosphatase and tensin homolog

Overall, a higher rate of patients died of disease in the PTEN-L population (*n =* 26, 9.4%, PTEN-L versus *n =* 8, 2.5%, PTEN-WT; *p* = 0.0001), particularly in HR−/HER2- (*p* = 0.0006) and locally advanced or metastatic breast cancers (*p* = 0.0006), as shown in Table 3. According to our multivariable model, PTEN status was an independent predictor of both death for disease and disease recurrence. In particular, loss of expression was significantly associated with patients’ death (*p* = 0.001) and the presence of unfavorable prognostic factors, such as triple-negative (*p* = 0.002) and HER2+ (*p* < 0.0001) phenotypes, as depicted in Table 4. Not surprisingly, patients with HR−/HER2- breast cancer harbored a high risk of death (*n =* 10, 14.3%; *p* = 0.0006) (Table 3). Of note, in PTEN-L tumors, HR−/HER2+ and HR+/HER2+ clusters showed an increased death prevalence (*n =* 1, 25.0% and *n* = 4, 26.7%, respectively) compared to the HR−/HER2- and HR+/HER2- (*n =* 4, 8.3% and *n* = 13, 6.1%, respectively; *p* = 0.02) (Fig. 3, Supplementary Table S2 and Table S3). These analyses provide evidence that PTEN loss or reduced expression is a bona fide prognostic parameter in breast cancer.

**Table 3:** Bivariate analysis showing the association of selected clinicopathologic characteristics with patients’ death

	Death		
	Yes	No	***p***-value
**HR and HER2 status, n (%)**			0.0006
HR+/ HER2-	18 (3.7)	465 (96.3)	
HR−/ HER2+	1(10.0)	9(90.0)	
HR+/ HER2+	5(12.0)	35(88.0)	
HR−/ HER2-	10 (14.3)	60 (85.7)	
**Stage, n (%)**			0.0006
0, 1	8 (3.0)	263 (97.0)	
2	12 (5.6)	204 (94.4)	
3, 4	15 (12.9)	101 (87.1)	
**PTEN status**			0.0001
PTEN-WT, n (%)	8 (2.5)	317 (97.5)	
PTEN-L, n (%)	26 (9.4)	252 (90.6)	

Death status was available for 603 patients. *PTEN-L* PTEN low (i.e. decreased expression), *PTEN-WT* PTEN wild-type (i.e. retained expression), *HR* Hormone receptors

**Table 4:** Multivariable analysis showing the association of selected clinicopathologic characteristics with PTEN status

PTEN-WT vs. PTEN-L			
	OR	95% CI	***p***-value
**Death**			
Survived vs. Deceased	0.25	0.11–0.55	0.001
**Grade**			
1/2 vs. 3	0.853	0.62–1.18	0.329
**HR and HER2 status**			
HR+/HER2- vs. HR+/HER2+	0.78	0.4–1.51	0.451
HR−/HER2- vs. HR+/HER2+	0.275	0.122–0.621	0.002
HR−/HER2+ vs. HR+/HER2+	0.9	0.22–3.72	0.884
HR−/HER2- vs. HR+/HER2-	0.36	0.21–0.61	< 0.0001
HR−/HER2+ vs. HR+/HER2-	1.16	0.32–4.17	0.818
HR−/HER2+ vs. HR−/HER2-	3.27	0.84–12.78	0.09

*PTEN-L* PTEN low (i.e. decreased expression), *PTEN-WT* PTEN wild-type (i.e. retained expression), *HR* Hormone receptors, *OR* Odds ratio, *CI* Confidence interval

### Shorter survival in HR-negative PTEN-L breast cancers

**Fig. 4:**
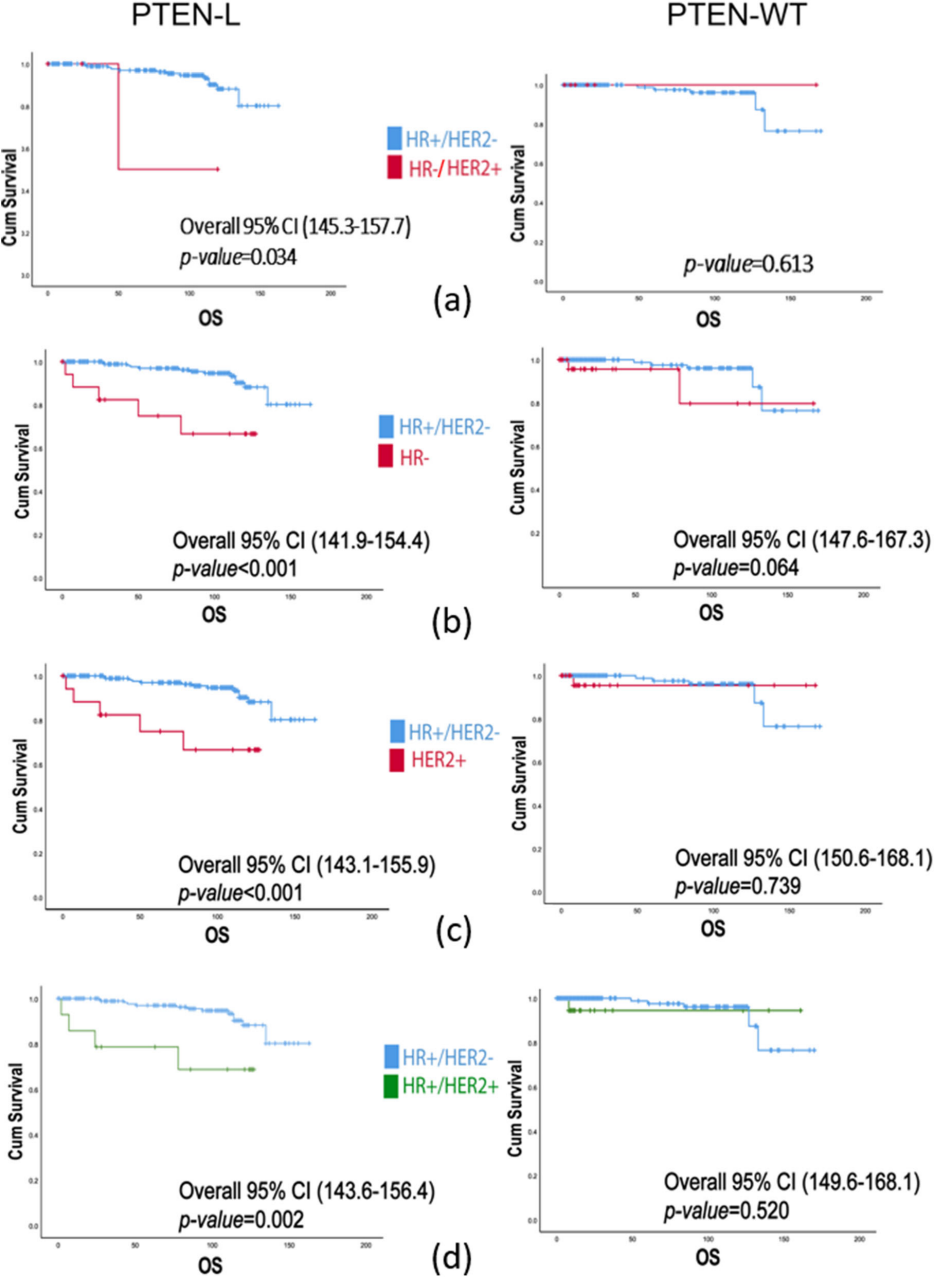
Survival analysis according to the combined status of PTEN, HR, and HER2. The Y-axis shows the cumulative survival while the X-axis represents the months of overall survival. HR, hormone receptor; (OS) overall survival; (CI) confidence interval

Survival analysis stratified for PTEN status (Fig. 4, Supplementary Fig. S1, and Supplementary Fig. S2) showed that, patients with PTEN-L (*p =* 0.03) but not PTEN-WT (*p =* 0.61) and HR−/HER2+ breast cancer have a shorter survival probability compared to HR+/HER2- (Fig. 4a). This correlation was not retained while analyzing the risk of recurrence, which was not statistically significant in both PTEN-L and PTEN-WT groups (Supplementary Fig. S2a). Given the low number of patients with HR+/HER2+ breast cancers in both PTEN cohorts, we further assessed HR negativity and HER2 positivity, as solo, along with PTEN status (Fig. 4b, c and Supplementary Fig. S2b, c). In both cases, the OS but not the disease-free survival was worse in PTEN-L compared to PTEN-WT neoplasms (*p* < 0.001 vs *p =* 0.06 and *p* < 0.001 vs. *p =* 0.73). Moreover, we observed that HER2 positivity either alone, or alongside with HR positivity, was associated with an increased risk of death in PTEN-L breast cancers (*p* < 0.0001 and *p* = 0.002, respectively), as shown in Fig. 4. Accordingly, none of these conditions were related to a worse prognosis when the expression of PTEN was retained (*p* = 0.73 and *p* = 0.52, respectively). Taken together, these findings suggest that patients with HR- and/or HER2+ breast cancer have an unfavorable prognosis in terms of OS in the presence of low or null expression of PTEN but not if PTEN expression is retained.

## Discussion

Here, we analyzed the PTEN status to assess its usefulness for the refining of breast cancer risk profiles in combination with traditional biomarkers testing. Our analyses show that a decreased expression of PTEN at the protein level occurs in almost half of patients, with the highest frequency in ductal and HR+ breast cancers. On the other hand, alterations of gene expression are present in the minority of patients, as previously reported [31]. The different frequency of PTEN protein and gene alterations can be due to the several regulatory layers that mediate PTEN function, including transcriptional (e.g. epigenetic mechanisms and transcription factors), post-transcriptional (e.g. miRNAs, PTEN pseudogene), and post-translational mechanisms (e.g. phosphorylation, acetylation, ubiquitination, etc.) [6, 32, 33]. Furthermore, we confirm that in breast cancer a wide spectrum PTEN expression patterns can be observed, emphasizes the need for the implementation of well-defined IHC guidelines [34, 35].

Despite their well-defined predictive role for endocrine therapy [25, 36], HR have been traditionally considered weak prognostic biomarkers in breast cancer [37]. On the other hand, previous studies on endometrial cancer and pancreatic neuroendocrine tumors have unraveled a rationale for the combined assessment of HR and PTEN for patients’ risk stratification [38, 39]. We found that PTEN expression levels show a statistically significant correlation with HR and HER2 status in breast cancer. Specifically, the prevalence of the HR−/HER2- subgroup, was more than double in the PTEN-L compared to PTEN-WT group. Furthermore, we confirm a higher incidence of death and disease recurrence in the PTEN-L population. Notably, the combined assessment of PTEN with HR and HER2 status showed more precise risk profiles.

When PTEN expression was low, ER negativity and HER2 positivity were related to worse OS compared to the HR+/HER2- subgroup. Moreover, in the PTEN-L but not in the PTEN-WT population, the HR+/HER2+ subgroup showed a statistically significant worse OS compared to the HR+/HER2- cluster. One of the possible explanations for the different risks observed in HR- and HER2+ breast cancer according to the PTEN status can be related to the very biological functions of this tumor suppressor. Indeed, the PI3K/Akt/mTOR pathway is the most commonly upregulated pathway in HR- and HER2+ breast cancers, being involved in many aspects of cell growth, proliferation, survival, metabolism, and immune response regulation [40]. A deep disturbance of these processes is caused by abnormal activating events targeting PI3K/Akt, which leads to tumorigenesis, metastasis, tumor progression, and therapy resistance [36, 41–43]. In addition, PTEN activity in the nucleus is critical for tumor suppression due to the modulation of the DNA damage response and anti-tumor immune activity, independently of PTEN phosphatase activity [44–47]. These transversal biological roles might explain the implication of PTEN in the development of therapy resistance in breast cancer [48, 49]. On the other hand, it has been proposed that loss of PTEN expression might be related to selective therapeutic pressure [50]. A recent study conducted on ER+ advanced breast cancer patients treated with a combination of the CDK4/6 inhibitor ribociclib and letrozole showed that loss of PTEN expression due to AKT activation could lead to the development of resistance to CDK4/6 inhibition [51]. Along with the observation that PTEN loss promotes resistance to PI3Kα inhibitors, the authors highlight the possibility that one genetic event might prove sufficient for the same patient to develop clinical cross-resistance to multiple therapies, including anti-HER2 and ET [51].

This study has intrinsic limitations. First, given its retrospective nature and the long timeframe of patients’ recruitment, it was not possible to unform the cohort for the treatment received. This could have led to an overestimation of PTEN independence as a prognostic biomarker, particularly in the trastuzumab-treated HER2 cohort. Indeed, a small percentage of patients with HER2+ breast cancer may not have received targeted adjuvant therapy, as before 2006 it was not approved in our Institution. Therefore, we cannot rule out the possibility that the lack of correlation of PTEN-WT and worse prognosis in HER2+ breast cancers could be related to a carry-over effect of the treatment with anti-HER2 drugs, in particular considering the different protocols adopted during. However, this correlation was statistically significant in the PTEN-L group. To this end, functional studies exploring the specific role of anti-HER2 drugs in PTEN-L breast cancer would be needed. Second, the relatively small number of tumors analyzed might have affected the ability to find additional correlations between PTEN and other significant clinicopathologic features. This study, however, should be considered hypothesis-generating. Further investigations in wider independent cohorts, with comprehensive molecular data and biostatistical analyses, are warranted to validate the clinical role of PTEN testing in breast cancer. Despite these limitations, this study offers novel insights on the potential clinical utility of combined PTEN, HER2, and HR testing for the identification of patients with high-risk breast cancer.

## Conclusions

In conclusion, decreased expression of PTEN at the protein level is seen in almost half of breast cancer patients. We found a positive correlation between PTEN protein expression with HR and HER2 status and by the decreased relative expression of PTEN, both HR- and HER2 overexpression/amplification were significantly related to worse OS compared to the HR+/HER2- status. Moreover, this HER2 positivity either alone or concomitantly with HR positivity was associated with poorer survival compared to the HR+/HER2- status. Hence, the combined analysis of PTEN, HR, and HER2 may provide additional data to perform a tailored risk assessment while evaluating patients with breast cancers.

## Supplementary Information

**Additional file 1: Figure S1.** Overall survival according to the PTEN status. **Figure S2.** Survival analysis according to the combined status of PTEN, HR, and HER2. The Y-axis shows the cumulative survival while the X-axis represents the months of disease-free survival.

**Additional file 2: Table S1.** List of antibodies, clones, dilutions, antigen retrieval methods, and scoring systems adopted for immunohistochemical analyses. **Table S2.** Bivariate analysis showing the association of combined assessment of PTEN-low and HR and HER2 markers with patients’ death. **Table S3.** The demographic and clinical characteristics of all PTEN, HR and HER2 expression comparison.

## Data Availability

The data presented in this study are available on request from the corresponding author. Publicly available datasets were analyzed in this study. This data can be found at https://www.cbioportal.org/.

## Abbreviations

PTEN: Phosphatase and tensin homolog
PI3K: Phosphatidylinositol-3-kinase
HR: Hormone receptors
BCIRG: Breast Cancer International Research Group
WHO: World Health Organization
AJCC: American Joint Committee on Cancer
FFPE: Formalin-fixed, paraffin-embedded
TMA: Tissue microarray
ER: Estrogen receptor
PgR: Progesterone receptor
CAP: College of American Pathologists
PTEN-L: PTEN-low
PTEN-WT: PTEN-retained
TCGA: The Cancer Genome Atlas Network
OR: Odds ratio
CI: Confidence interval
SD: Standard deviation
OS: Overall survival
