# Supplementary figures and images for: Combined analysis of PTEN, HER2, and hormone receptors status: remodeling breast cancer risk profiling
Source: BMC Cancer. 2021 Oct 28;21:1152. doi: 10.1186/s12885-021-08889-z (PMC8555186; doi:10.1186/s12885-021-08889-z)

Supplementary Figure 1

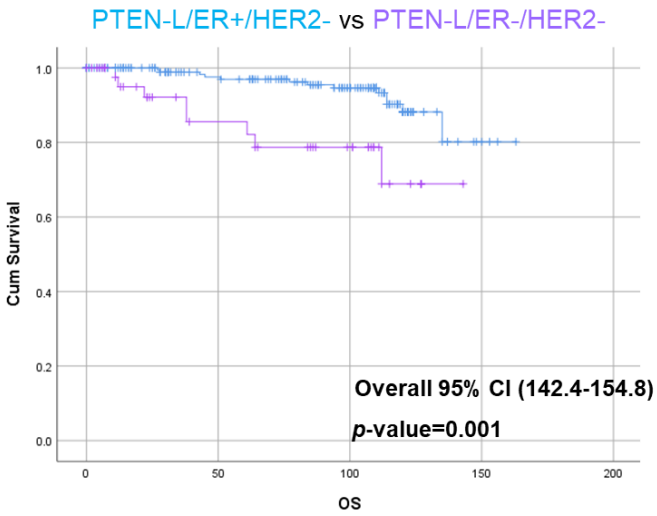

(a.1)

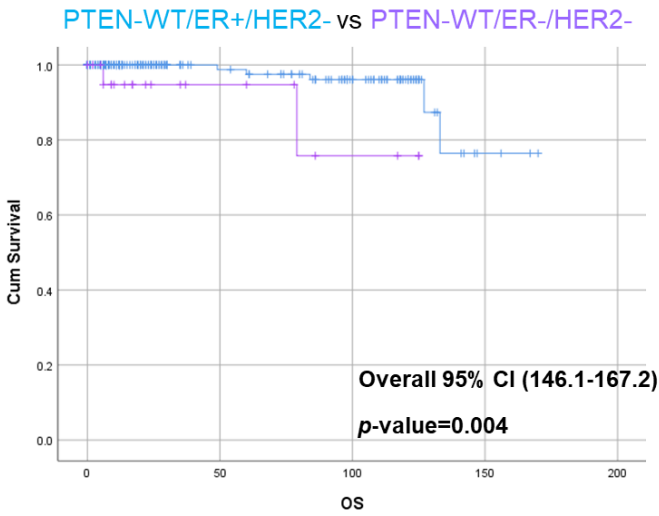

(a.2)

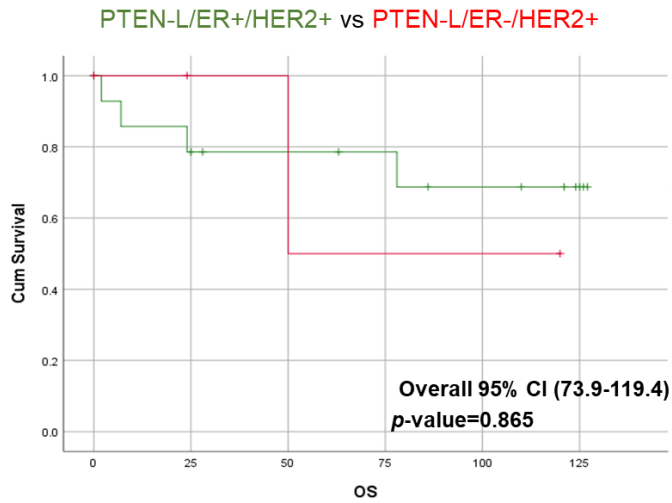

(b.1)

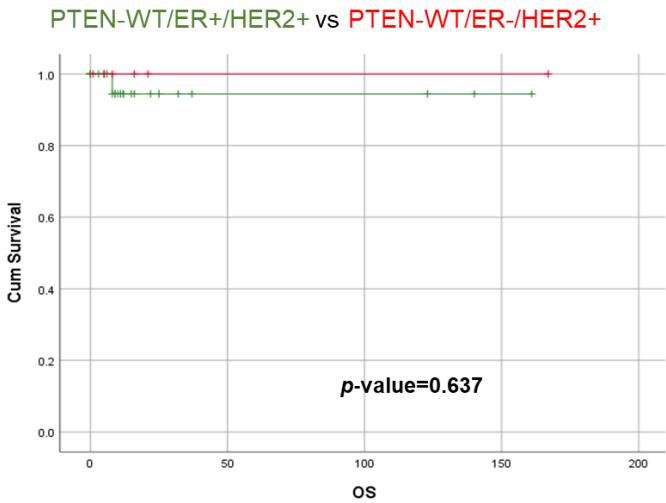

(b.2)

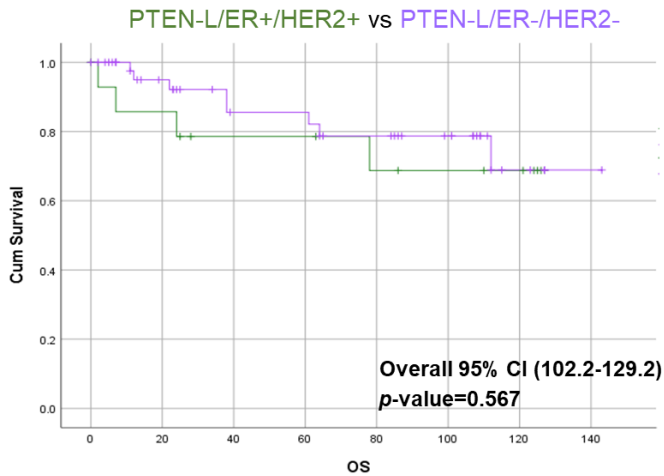

(c.1)

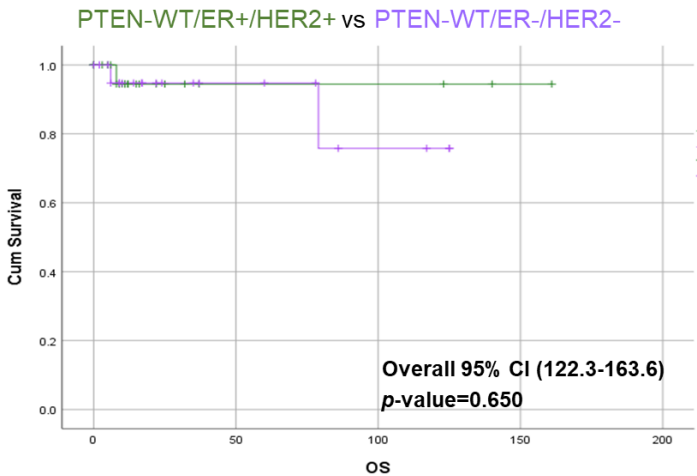

(c.2)

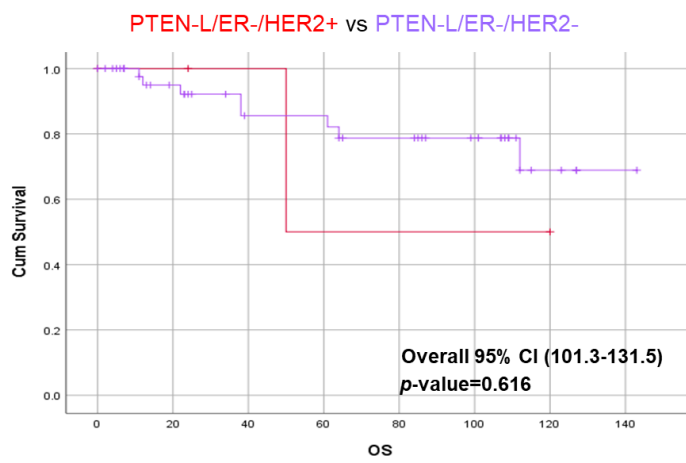

(d.1)

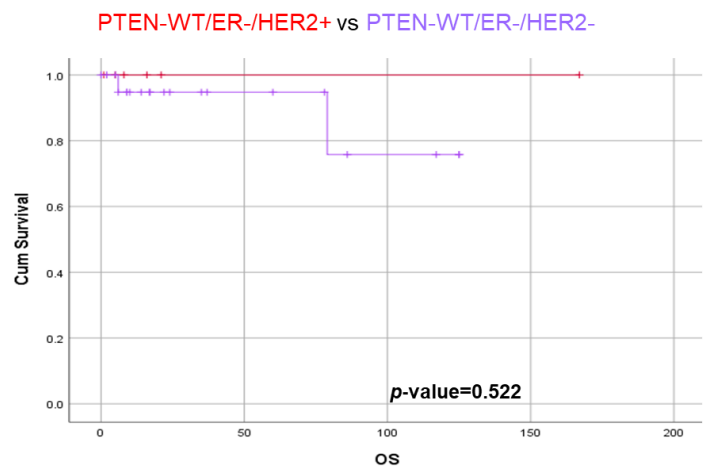

(d.2)

## Supplementary Figure 2

PTEN-L

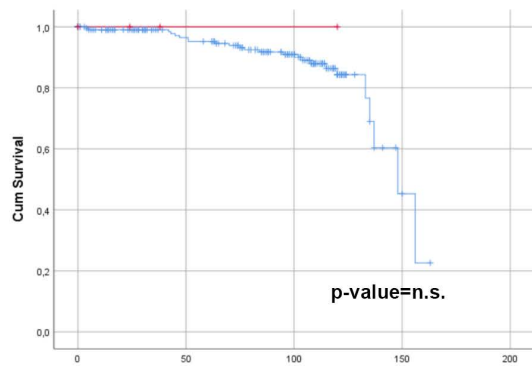

(a)

PTEN-WT

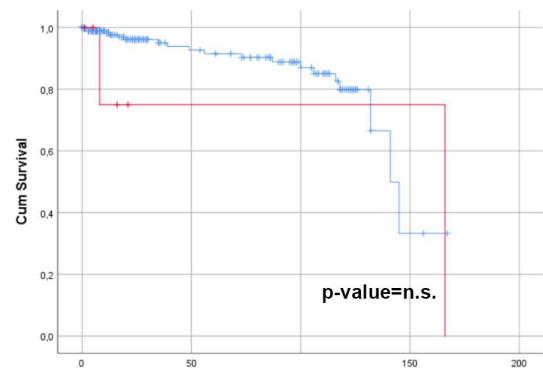

(b)

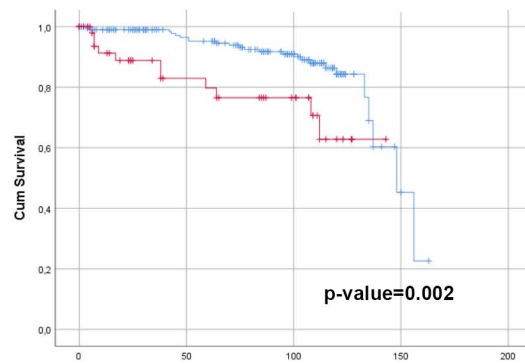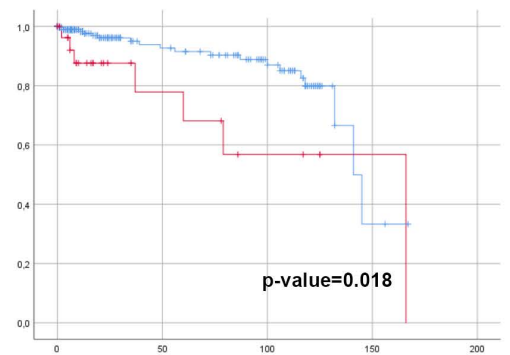

(c)

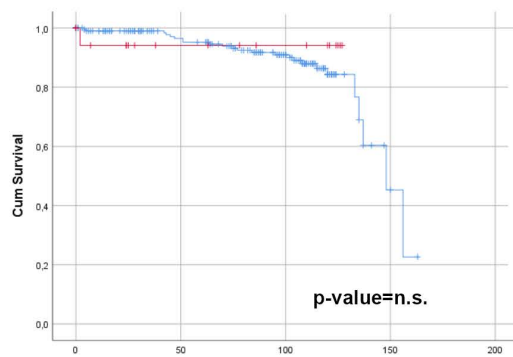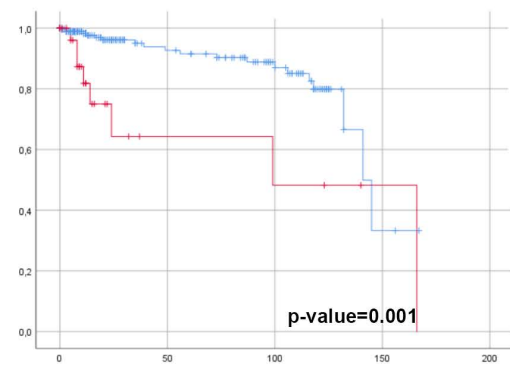

(d)

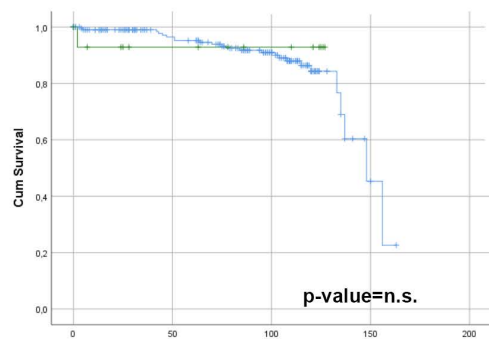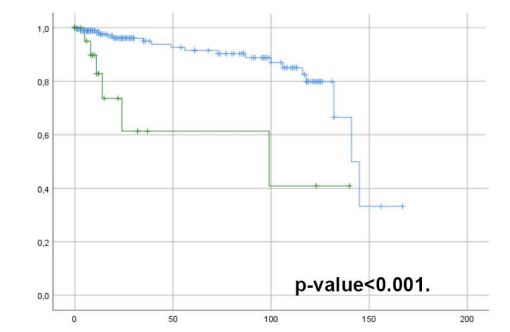

Supplement: Supplementary file 1 — Additional file 1: Figure S1. Overall survival according to the PTEN status. Figure S2. Survival analysis according to the combined status of PTEN, HR, and HER2. The Y-axis shows the cumulative survival while the X-axis represents the months of disease-free survival. [file 12885_2021_8889_MOESM1_ESM.pdf]
