# Supplementary material for: Combined analysis of PTEN, HER2, and hormone receptors status: remodeling breast cancer risk profiling
Source: BMC Cancer. 2021 Oct 28;21:1152. doi: 10.1186/s12885-021-08889-z (PMC8555186; doi:10.1186/s12885-021-08889-z)
Supplement: Supplementary file 2 — Additional file 2: Table S1. List of antibodies, clones, dilutions, antigen retrieval methods, and scoring systems adopted for immunohistochemical analyses. Table S2. Bivariate analysis showing the association of combined assessment of PTEN-low and HR and HER2 markers with patients’ death. Table S3. The demographic and clinical characteristics of all PTEN, HR and HER2 expression comparison. [file 12885_2021_8889_MOESM2_ESM.pdf]

SUPPLEMENTARY TABLES

Supplementary Table S1. List of antibodies, clones, dilutions, antigen retrieval methods, and scoring systems adopted for immunohistochemical analyses. ER, estrogen receptor alpha; PR, progesterone receptor; PTEN, Phosphatase and tensin homolog.

Marker	Clone	Dilution	Technology	Antigen retrieval	Scoring
ER	EP1	Ready to use	Dako Omnis	EnVision FLEX, High pH, 20'	ASCO/CAP and St Gallen guidelines; positive if ≥1% of tumor cell nuclei are immunoreactive, high if >20% of tumor cell nuclei are immunoreactive
PR	PgR 636	1:100	Dako Omnis	EnVision FLEX, High pH, 30’	ASCO/CAP and St Gallen guidelines; positive if ≥1% of tumor cell nuclei are immunoreactive, high if >20% of tumor cell nuclei are immunoreactive
Ki67	MIB1	Ready to use	Dako Omnis	EnVision FLEX, High pH, 30’	ASCO/CAP and St Gallen guidelines; high if >30% of tumor cell nuclei are immunoreactive
HER2	Polyclonal	1:400	Dako Omnis	EnVision FLEX, Low pH, 30'	ASCO/CAP guidelines; 3+ if uniform intense membrane staining circumferential membrane staining that is complete and intense, 2+ if circumferential membrane staining that is incomplete and/or weak/moderate and within >10% of the invasive tumor cells or complete and circumferential membrane staining that is intense and within ≤10% of the invasive tumor cells, negative for other staining patterns
PTEN	6H2.1	1:100	Dako Omnis	EnVision FLEX, High pH, 30'	Retained expression: score 2 (staining in tumor cells equal to normal ductal and stromal cells);<br>Low expression: score 1 (staining in tumor cells weaker than normal ductal and stromal cells) or score 0 (staining absent in tumor cells but present in normal ductal and stromal cells).

Supplementary Table S2. Bivariate analysis showing the association of combined assessment of PTEN-low and HR and HER2 markers with patients’ death. HR, estrogen receptor; PTEN, phosphatase and tensin homolog.

	Death		
	**Yes**	**No**	***p*-value**
**PTEN-L subgroups, n (%)**			0.02
HR+/ HER2-	13 (6.1)	200 (93.9)	
HR-/ HER2-	4 (8.3)	44 (91.7)	
HR-/ HER2+	1 (25.0)	3 (75.0)	
HR+/ HER2+	4 (26.7)	11 (73.3)	

Supplementary Table S3. The demographic and clinical characteristics of the breast cancer clusters based on the PTEN status.

	**PTEN-WT**				**PTEN-L**			
	**HR+/HER2-**	**HR-/HER2-**	**HR-/HER2+**	**HR+/HER2+**	**HR+/HER2-**	**HR-/HER2-**	**HR-/HER2+**	**HR+/HER2+**
**Grade**								
G1	40	0	0	0	28	3	0	1
G2	138	1	0	5	102	6	1	5
G3	97	21	6	20	83	39	3	9
**Stage**								
I	136	6	2	10	93	18	2	5
II	92	11	1	8	80	21	1	5
III	42	5	3	6	38	8	1	5
VI	5	0	0	1	2	1	0	0
**Death**								
No	269	20	6	24	200	44	3	11
Yes	6	2	0	1	13	4	1	4
**Total**	275	22	6	25	213	48	4	15
